# Exposure to Extremely Partisan News from the Other Political Side Shows Scarce Boomerang Effects

**DOI:** 10.1007/s11109-021-09769-9

**Published:** 2022-02-03

**Authors:** Andreu Casas, Ericka Menchen-Trevino, Magdalena Wojcieszak

**Affiliations:** 1 Vrije Universiteit Amsterdam, Amsterdam, The Netherlands; 2 American University, Washington, United States of America; 3 University of California - Davis, Davis, United States of America; 4 University of Amsterdam, Amsterdam, The Netherlands

**Keywords:** Polarization, Counter-attitudinal information, Boomerang effect, Backfire effect

## Abstract

A narrow information diet may be partly to blame for the growing political divides in the United States, suggesting exposure to dissimilar views as a remedy. These efforts, however, could be counterproductive, exacerbating attitude and affective polarization. Yet findings on whether such boomerang effect exists are mixed and the consequences of dissimilar exposure on other important outcomes remain unexplored. To contribute to this debate, we rely on a preregistered longitudinal experimental design combining participants’ survey self-reports and their behavioral browsing data, in which one should observe boomerang effects. We incentivized liberals to read political articles on extreme conservative outlets (Breitbart, The American Spectator, and The Blaze) and conservatives to read extreme left-leaning sites (Mother Jones, Democracy Now, and The Nation). We maximize ecological validity by embedding the treatment in a larger project that tracks over time changes in online exposure and attitudes. We explored the effects on attitude and affective polarization, as well as on perceptions of the political system, support for democratic principles, and personal well-being. Overall we find little evidence of boomerang effects.

## Introduction

Political polarization is a problematic feature of many societies. Partisans hold increasingly disparate positions (Newport & Dugan, 2017) and are becoming hostile toward their political opponents (Iyengar et al., 2019). This problem is particularly relevant in the context of the ongoing debate about online echo chambers, which are said to polarize individual views and lead to outgroup hostility, (Garrett, 2009; Pariser, 2011; Sunstein, 2011) and the discussion about the potential depolarizing benefits of a diverse media diet (Stroud, 2010; Helberger, 2012). Democratic theorists have long argued that encountering opposing arguments on issues of the day should promote “representative thinking” (Arendt 1968, p. 241), “sound political judgment” (Page, 1996, p. 2), and “enlightened understanding” (Dahl, 1989, p. 105), and transform citizens into a cohesive collective (Barber, 1984), outcomes that point to dissimilar exposure as a remedy to polarization. Accordingly, stakeholders such as social media companies (Farr & Dorsey, 2018), news organizations (Goodman & Chen, 2010), public agencies and governments (Rendall, 2015) are working on reducing ideological bubbles. Evaluating whether exposure to counter-attitudinal perspectives indeed achieves the intended results is crucial for designing strategies that can effectively reduce polarization.

Theoretically, political psychology suggests that counter-attitudinal exposure should generate a boomerang effect, strengthening people’s existing policy views—*attitude polarization*—and generating negative feelings towards political outgroups—*affective polarization* [see e.g., (Taber et al., 2001; Taber & Lodge, 2006)]. Existing evidence is inconclusive: some find polarization following counter-attitudinal exposure (Garrett et al., 2014; Wojcieszak, 2011; Zhou, 2016; Bail et al., 2018; Taber & Lodge, 2006), yet these worrying effects are not borne out in other studies (Beam et al., 2018; Levy, 2021; Guess & Coppock, 2018), leading some scholars to argue that boomerang effects “are the exception, not the rule” (Guess & Coppock, 2018, p. 4).^1^ Given the potential large-scale implications of these types of interventions, it is crucial to establish whether or not (and for whom) counter-attitudinal exposure can backfire.

Furthermore, whereas polarization is arguably important, exposure to counter-attitudinal views can have effects on broader systemic outcomes that go beyond issue attitudes or feelings toward political opposition, such as trust in the political system or support for key democratic principles, and also on individual well-being and health. If we find that counter-attitudinal exposure attenuates polarization, but also minimizes political trust or diminishes people’s well being for instance, should we promote it?

In this paper, we offer both theoretical and methodological advancements. Theoretically, we test the boundary conditions of the boomerang effect (i.e., creating an encouragement design, in which this effect should emerge) and assessing the effects of counter-attitudinal exposure on a wide range of relevant societal and individual-level outcomes. Methodologically, we rely on an over-time encouragement design in a naturalistic setting, extending past work that tested short-term effects from one-shot exposure to stimuli (Taber and Lodge, 2006; Zhou, 2016) and adding to recent innovative tests of boomerang effects (Bail et al., 2018; Guess, 2021; Levy, 2021). We additionally incorporate rich qualitative responses to contextualize our findings. Specifically, we combine experimental data with online behavioral traces from the same participants, with the aim of testing a set of pre-registered hypotheses^2^ regarding the consequences of counter-attitudinal exposure on attitude and affective polarization, as well as on a broader set of outcomes. We constructed a stimulus that, while unlikely to occur in the real world, is perfectly suited for the goal at hand. For a two week period, we incentivized liberals to consume content from extremely conservative sites (Breitbart, The American Spectator, and The Blaze), and incentivized conservatives to consume content from extremely liberal sites (Mother Jones, Democracy Now, and The Nation). If in this scenario people do *not* polarize, then we have strong evidence that boomerang effects are the exception and not the norm. If people do polarize, however, this counter-factual allows us to understand the boundary conditions of boomerang effects on attitude and affective polarization, as well as on other relevant systemic and individual outcomes. We also test whether our treatment has different effects depending on one’s political priors (i.e., partisanship and ideology strength, political identity strength) and we also explore these effects among Republicans and Democrats to speak to concerns about asymmetric polarization (Grossmann & Hopkins, 2016).

First, we examine whether consuming extreme sites of the opposing ideology increases the extremity of people’s attitudes on five salient issues: the economy, climate change, gun control, immigration, and the Presidency of Donald Trump (i.e., attitude polarization). Our results align with with Guess and Coppock (2018)’s and Levy (2021)’s findings: we do not observe people’s policy views becoming more extreme; a finding that also holds for those with stronger political identities (see also Wojcieszak et al. (2021a)).

In addition, we test the effects of our treatment on changes in affective polarization toward a range of political outgroups: supporters of the opposing party, those of opposing ideology, and those holding opposing views on the five aforementioned issues. We find no increases in affective polarization towards out-partisans and out-ideologues, no matter the level of respondents’ party and ideological strength. Although exposure to extreme sites from the opposing side led to minor increases in hostility towards those holding different views on some policy issues, this effect disappeared when accounting for multiple comparisons. Finally, in order to speak to the overall consequences of (extreme) dissimilar exposure, we assess an additional set outcomes (i.e. attribution of malevolence, support for compromise, perceived polarization, trust in key societal institutions, support for freedom of speech and the press, as well as participant’s well-being). As above, we find a consistent pattern of null effects.

We guard against several threats to our conclusions (e.g., attrition bias), account for differential levels of compliance (measured using both self-reported and online behavioral data), and contextualize our results with open-ended qualitative data. In sum, we conclude that there is little evidence that exposure to counter-attitudinal content (here, to extreme news sites of the opposing ideology) exacerbates polarization or other relevant outcomes.

## Are There Boomerang Effects?

In the US and internationally, political divisions are on the rise. The gap between issue attitudes of the left and the right is growing (Newport & Dugan, 2017), as is affective polarization: Democrats dislike the Republicans and vice versa, attribute negative traits to the out-party, and avoid social interactions with its members (Iyengar et al., 2012, 2019; Chen & Rohla, 2018). A prevalent line of scholarship points to partisan media and narrow media diets as key determinants of polarization (Sunstein, 2011). Consumption of partisan news or hyper-partisan content can radicalize issue attitudes (Knobloch-Westerwick & Meng, 2011; Levendusky, 2013) and increase hostility toward the political outgroup (Garrett et al., 2014; Wojcieszak et al., 2020), effects that can spread to those who do not consume partisan news directly (Druckman et al., 2018).^3^ It follows that cross-cutting flows of information could be a remedy to polarization (Mutz, 2002; Nelson, 2015; Wojcieszak et al., 2020). Democratic theorists have long argued that diverse exposure is crucial for a healthy, respectful, and sustainable democracy (Arendt, 1968; Barber, 1984; Page, 1996) and some work on cross-cutting networks and media use suggests that encountering different viewpoints has the potential to moderate people’s attitudes and feelings towards out-groups (Mutz, 2002; Nelson, 2015; Wojcieszak & Warner, 2020).

That said, established theoretical frameworks on information processing and public opinion formation (e.g., confirmation bias, motivated reasoning (Kunda, 1990; Redlawsk, 2002; Taber & Lodge, 2006)), the Receive–Accept–Sample model (Zaller, 1992); see Guess and Coppock (2018) for a review) suggest that exposing people to counter-attitudinal information can exacerbate political divides. Rather than objectively weighing the pros and cons of an argument in order to form a correct belief, people desire to maintain their priors. This process should result in polarization (Lord et al., 1979; Taber & Lodge, 2006). Indeed, when exposed to opposing views, from the media and during online or face-to-face discussions, people end up more extreme (Wojcieszak & Price, 2010; Zhou, 2016; Levendusky, 2013; Bail et al., 2018) and more hostile toward various social out-groups (Wojcieszak, 2011). For instance, Levendusky (2013) exposed subjects to counter-attitudinal clips from FoxNews (for liberals) and MSNBC (for conservatives), finding that those with strong pre-treatment attitudes radicalized their views. Also, Bail et al. (2018) found that conservative (not liberal) Twitter users polarized after one month of following a bot sharing 24 messages a day from out-group political elites.

However, other work fails to replicate these results, mostly finding no boomerang effects from counter-attitudinal exposure. As an example, Guess and Coppock (2018) conducted three experiments to see if people’s opinions about contentious issues radicalized after exposure to counter-attitudinal information. They did not find that to be the case, concluding that boomerang effects are the exception rather than the norm. In a recent field experiment, Guess et al. (2021) combined web tracking data with an encouragement design asking participants to change the homepage on their browsers to Fox News or HuffPost, follow the source’s Facebook page, and subscribe to affiliated newsletters for one month. Induced exposure to partisan media had no polarizing effects. And yet Levy (2021), who randomly assigned Facebook users to subscribe to up to four liberal or conservative outlets on Facebook (e.g., MSNBC or Fox News), finds that exposure to counter-attitudinal news decreased affective polarization and had no effects on policy attitudes (for null effects see also Wojcieszak et al. (2021a)).

Hence, despite its theoretical relevance and practical implications, the debate about the boomerang effects is far from settled. The inconsistencies in extant evidence may be due to a variety of factors, such as differing methodologies and designs (e.g., immediate pre-post test forced exposure experiments, field experiments), variations in samples (e.g., students, participants in online panels, social media users), treatments (e.g., news clips, tweets, homepages), platforms (Facebook, Twitter), issues (e.g., general politics, specific policies/issues), among others. Our goal is not to pinpoint the reasons for the inconsistencies nor resolve the debate in any final or conclusive way, as no single project can do so and a meta-analysis accounting for these various factors is needed.

Our three objectives are more modest. First, we hope to advance the debate about boomerang effects by testing their boundary conditions in an encouragement design, in which these effects are most likely to emerge. Second, we offer more nuance and shed light on the reasons behind the results by incorporating qualitative data to contextualize the quantitative findings, to our knowledge, the first study of this kind. Lastly, we aim to better understand any further societal and individual effects of exposing people to counter-attitudinal news sources. Although existing studies have evaluated the consequences of such exposure on polarization, little is known about its societal (e.g., trust in key institutions) and individual (e.g., well-being) implications.

## Outcomes and Hypotheses

### Attitude Polarization

Most work on boomerang effects studies changes in policy attitudes looking at attitude strength (Zhou, 2016), and/or extremity (Wojcieszak, 2010; Levendusky, 2013; Guess & Coppock, 2018; Bail et al., 2018). We focus on attitude extremity, so the extent to which people radicalize their views about policy issues, an outcome of relevance given that divergent policy views are often blamed for government gridlock (Lee, 2015).

As aforementioned, literature finds mixed results on whether *in general* people’s attitudes become more extreme after counter-attitudinal exposure (Guess & Coppock, 2018; Bail et al., 2018). Yet, the motivated reasoning literature predicts boomerang effects particularly among those with strong predispositions (Lord et al., 1986; Taber & Lodge, 2006). Because most people do not hold clear policy position and do not follow politics (Converse, 1964; Carpini & Keeter, 1996; Hibbing, 2002), boomerang effects are unlikely to emerge in the aggregate and—theoretically—those with stronger priors should polarize their attitudes after counter-attitudinal exposure. Our first set of pre-registered hypotheses therefore predicted:

$${H} _{1a}$$
Participants exposed to extreme news sites of the opposing ideology will not polarize their policy attitudes.
$${H} _{1b}$$
Participants with stronger political identities will be more likely to polarize their policy attitudes when exposed to extreme news sites of the opposing ideology.

### Affective Polarization

Beyond attitude extremity, increasing animosity between political groups also thwart consensual democracy (Iyengar et al., 2019). Despite not paying much attention to politics (Hibbing, 2002), most people feel attached to political groups (Campbell 1980) and interpret day-to-day politics using an us-*versus*-them logic (Tajfel et al., 1979). Exposure to counter-attitudinal information may make in/out-group conflicts more salient, increasing people’s negative feelings toward out-groups. Such in/out-group distinction should be more clear to those with stronger political group attachments.

$${H} _{2a}$$
Participants exposed to extreme news sites of the opposing ideology will hold more negative feelings towards members of political out-groups.
$${H} _{2b}$$
Participants with stronger political identities will be more likely to hold more negative feelings towards out-group members when exposed to extreme news sites of the opposing ideology.

### Perceptions of the Political System

A positive perception of the political system contributes to stability (Marien & Hooghe, 2011; Agroskin et al., 2015). We evaluate the effect of our treatment on five systemic indicators: (a) attribution of malevolence (i.e. whether the out-party wants to harm the country), (b) support for compromise (i.e. whether politicians should be open to compromise), (c) perceived polarization (i.e. seeing the political system as polarized), (d) people’s trust in a set of institutions, and (e) support for two democratic principles, freedom of speech and freedom of the press. As above, we expect exposure to extreme dissimilar domains to activate the presence of political conflict and to make people more pessimistic about the political system. We also expect more pronounced effects among those with stronger political identities/attachments.

$${H}_{3a}$$
Attribution of malevolence: Participants exposed to extreme news sites of the opposing ideology (${H}_{3b}$ especially those with stronger political identities) will be more likely to believe that the out-party want to harm the country.
$${H}_{4a}$$
Support for compromise: Participants exposed to extreme news sites of the opposing ideology (${H}_{4b}$ especially those with stronger political identities) will be less likely to support political compromise.
$${H}_{5a}$$
Perceived polarization: Participants exposed to extreme news sites of the opposing ideology (${H}_{5b}$ especially those with stronger political identities) will be more likely to perceive the political climate as polarized.
$${H}_{6a}$$
Trust in institutions: Participants exposed to extreme news sites of the opposing ideology (${H}_{6b}$ especially those with stronger political identities) will be less likely to trust key societal institutions.
$${H}_{7a}$$
Support for democratic principles: Participants exposed to extreme news sites of the opposing ideology (${H}_{7b}$ especially those with stronger political identities) will be less likely to endorse freedom of speech and of the press.

### Subjective Well Being

Preserving people’s well being is a desirable goal from a normative and humanitarian perspective. News consumption can generate emotional discomfort (Valentino et al., 2008), especially when people are exposed to information challenging their prior views. For example, Marcus et al. (2000) find people to feel more anxious when consuming news about the negative electoral prospects of their party. Building on political psychology models such as Marcus et al. (2000)’s “affective intelligence,” and on the literature on motivated reasoning (Taber & Lodge, 2006), we expect exposure to extreme opposing opinions to worsen how people feel (i.e. more anxious and less happy) and to increase behaviors induced by anxiety (i.e. consuming alcohol and/or junk food) or anger (i.e., getting into arguments or wanting to hit someone):

$$\text {H}_{8a}$$
The well being of participants (${H}_{8b}$ particularly those with stronger political identities) will worsen after exposure to extreme domains from the other side.

## Research Design

**Fig. 1:**
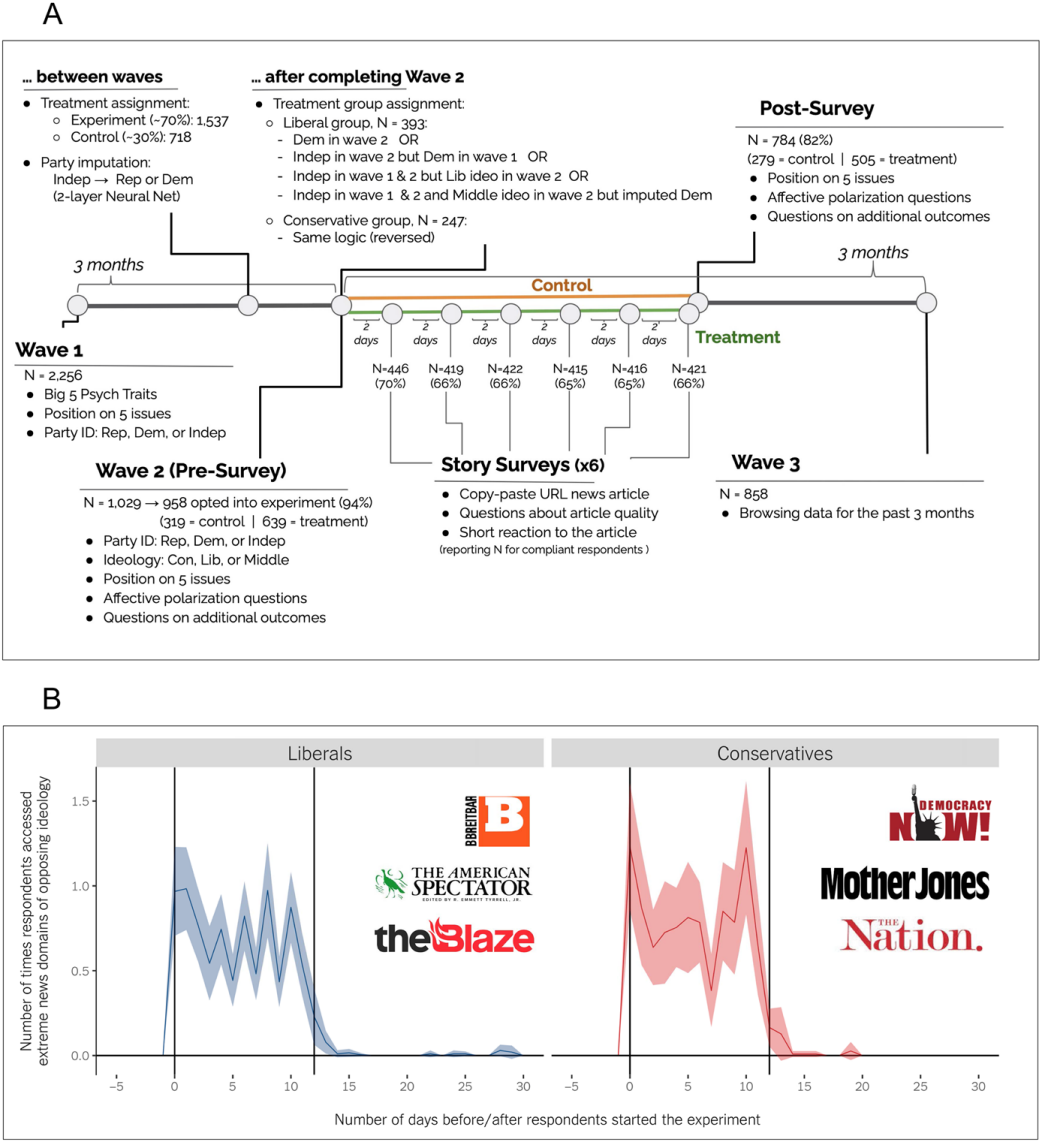
**A** Outline of the research design. **B** Average number of times the treated respondents accessed the news sites used in the study; before, during and after the experiment

Figure 1.A provides an overview of the design. We embedded our experiment in Wave 2 of a 3-wave panel study in which, every three months, the same respondents answered a 20-minute survey about their political views and news diet, and submitted their web browsing data using an open source browser plug-in that allows for transparent data sharing (https://www.webhistorian.org/ Web Historian). We recruited respondents via Lucid, an aggregator of survey respondents, which collects demographic information on the panelists, facilitating quota sampling to match the US Census margins. Before inviting them to participate in Wave 2, the 2256 respondents who completed Wave 1 were assigned to a treatment (with a 70% probability) or a control group. After completing Wave 2, i.e., after providing pre-treatment values for the variables of interest, respondents were invited to an additional study “on the quality of news” (this experiment).^4^ Out of the 1029 that completed Wave 2, 958 (94%) opted to take part in the experiment. To guard against the threat that those who opted in systematically differ on key variables from those who did not, in Sect. 13 we provide sociodemographic statistics for those who completed Wave 2 and for the subset who agreed to participate in the experiment. The two sets of respondents hold highly similar pre-test values on key characteristics (age, gender, education, ethnicity, party ID, ideology, and issue positions).

At this point, the treatment group (N = 639, *v.* 319 in the control group) was assigned to either a liberal (N = 393) or a conservative group (N = 247) based on participants’ pre-treatment partisanship.^5^

**Fig. 2:**
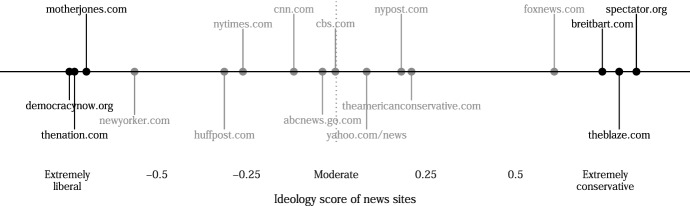
Ideology scores for the sites used in this experiment, as well as for other mainstream sites based on Robertson et al. (2018)

Then, for twelve days, those in the treatment groups were instructed to increase exposure to (very) dissimilar news sources. To further enhance external validity, we selected real news domains rather than creating mock news websites as done in some other work. Every other day, participants were asked to access one of the three domains of the opposing ideology (Breitbart, The American Spectator, and The Blaze for liberals; Mother Jones, Democracy Now, and The Nation for conservatives) and read an article on a salient political topic. As shown by the validated ideology scores (Robertson et al., 2018) in Fig. 2, these six outlets represent the most extreme ideological spaces in the media environment and are *equally extreme on each side*. Given that people do not often consume news (Wojcieszak et al., 2021b), and that when doing so, they rarely visit partisan websites (Guess, 2021; Wojcieszak et al., 2021a), we believe that visiting these extreme sites six times during a two-week period constitutes a rather strong treatment relative to one-shot exposure tested in past work (Levendusky 2013), but not relative to (less externally valid) massive exposure to tweets by opposing party elites tested in Bail et al. (2018).

To assure compliance, participants were instructed to copy-paste the URL of the article, write a brief reaction to the article, and answer two questions about the quality of the article. We use their answers to these six “story surveys” to measure compliance. The story surveys also allow us to better understand participants’ reactions to the treatment, particularly the open-ended question where they were asked to describe their reaction to the article. Because participants could have visited non-political articles, we rely on a stringent definition of compliance. A respondent is considered to have complied if: (a) the provided URL links to one of the assigned extreme news sites, (b) the article is about a *political* topic, and (c) they wrote a response to the article (to increase the chances participants read the article).^6^ We coded all the visited URLs for whether they existed and were about politics (and not, say, sports on Breitbart). Visits to non-political articles were *excluded* and not counted towards compliance. The control group did not engage in any additional activity. As another compliance check, we take advantage of the unique opportunity to return to the same respondents three months later as part of Wave 3 of data collection for the main project. Before Wave 3, participants provided 3 months of online browsing data stored on their computers,^7^ allowing us to examine the domains and URLs visited during the experiment. As Fig. 1.B illustrates, on average, the treated respondents did access the extreme sites at the expected rates (once every other day, so between 0.5 and 1 a day during the experiment). Importantly, the trace data additionally reveal that on average respondents spent 2 min and 40 s reading the news URLs they pasted in the story surveys. In stark contrast, the average time of a next visit across all news sites was only 40 s. In sum, we are confident that the participants were indeed treated as expected.

Out of the 639 respondents who opted into the experiment and were assigned to the treatment group, the following number completed and *fully complied* with each story survey: (1) 446 (70%); (2) 419 (66%), (3) 422 (66%), (4) 415 (65%), (5) 416 (65%), and (6) 421 (66%). As above, to guard against attrition bias, we compare these various sub-groups on key characteristics and observe no relevant differences (see Sect. 13).

After twelve days, both treatment and control groups were invited to complete a post-test survey that assessed the tested outcomes. In total, 279 respondents in the control and 505 in the treatment group completed the post survey, constituting the final sample for the study. In Sect. 13 we do not observe any concerning significant attrition bias when comparing those in the treatment group who completed the post survey to the control group.^8^

## Measures

We present question wording, Cronbach alphas, and descriptive statistics for all the measured items in Sect. 10. In Sect. 9 we also offer additional information on the items that comprised the attitude extremity scale.

### Attitude Extremity

To test $H_{1a}$ and $H_{1b}$, the pre and post survey measured respondents’ attitudes on five policy issues (economy, climate change, gun policy, immigration, and the Presidency of Donald Trump) using fifteen questions, three per issue, and asking subjects to locate their position in between a liberal or conservative statement at each end of a 13-point continuum.^9^ Table 1 provides the statements, paraphrased, and Sect. 9 contains the exact wording and scale properties. We average the responses per issue and report changes in attitudes towards the five issues.^10^

**Table 1:** Policy dimensions measured in the pre and post surveys

Reference	Policy dimension
Econ-1	*More* (v. less) government regulation of business
Econ-2	*More* (v. less) taxes to pay for public services
Econ-3	*Less* (v. more) free trade
Env-1	*More* (v. less) strict environmental regulation
Env-2	Human action *has* (v. has not) caused global warming
Env-3	US *should* (v. should not) emphasize alternative energy
Immig-1	Immigrants *strengthen* (v. weaken) the country
Immig-2	Illegal immigrants *should* (v. should not) be able to stay
Immig-3	Immigration *enriches* (v. impoverishes) American identity
Gun-1	*More* (v. less) regulation for buying a firearm
Gun-2	Banning the sale of semi-automatic weapons *will* (v. will not) prevent mass shootings
Gun-3	Concealed carriage *should not* (v. should) be allowed anywhere
Trump-1	Trust Donald Trump *less* (v. more) than other presidents
Trump-2	President Trumps respects white and men *more* (v. equally) than women and minorities
Trump-3	Trump’s presidency has been *bad* (v. good) for the economy

In italics, we indicate the position describing the *liberal* end of the scale, and show the conservative position in parentheses

### Affective Polarization

To test hypotheses $H_{2a}$ and $H_{2b}$, we measure affective polarization towards out-partisans (Republicans or Democrats), out-ideologues (conservatives or liberals), and those who hold different positions on the five issues. Each measure captures a slightly different and very relevant aspect of negative outgroup attitudes. We rely on the classic 100-point feeling thermometers (how warm people feel toward the out-group) and negative trait ratings (Iyengar et al., 2012) (how much respondents agree that outgroup members are ‘stupid’ or ’mean’). Because one may dislike the outgroup but nevertheless understand its perspectives, we measure how much respondents understand the views of outgroup members (7-point scale). In addition to accounting for different facets of affective polarization, using multiple measures also ensures that the detected patterns are not due to any specific measurement alone and that the results are robust to contexts and outgroups.

### Perception of the Political System

*Attribution of malevolence* ($\text {H}_{3a}$ and $\text {H}_{3b}$) we average the responses to five questions asking respondents to indicate how much they think that the opposing party wants to hurt the country (Warner & Villamil, 2017). *Support for compromise* ($\text {H}_{4a}$ and $\text {H}_{4b}$) we average the answers to four statements (found to be valid and reliable in Wave 1) regarding whether Republicans and Democrats should work together. *Perceived polarization* ($\text {H}_{5a}$ and $\text {H}_{5b}$) we average the responses to four questions asking subjects how much they perceive the political climate as polarized (also validated during wave 1). *Trust in institutions* ($\text {H}_{6a}$ and $\text {H}_{6b}$) we average the responses to questions about subjects’ trust in six societal institutions: three known to be more trusted by Republicans (Federal Government, the Supreme Court, and the police) and three more trusted by Democrats (scientists, journalists, and university professors) (Pew, 2017). *Support for freedom of speech* following Mutz (2002), respondents indicated the extent to which members of the opposing ideology should be allowed (a) in the media, (b) to make public speeches, (c) to hold public rallies, and (d) to teach in schools. *Support for freedom of the press* we average three responses about the extent to which (a) some media outlets should be made illegal, (b) Google should not show articles from some media outlets, and (c) social media companies should avoid promoting articles from some media outlets. The two last batteries were only asked in the post survey and so we use between-group difference to test hypotheses $\text {H}_{7a}$ and $\text {H}_{7b}$.

### Subjective Well Being

Six questions asked about the extent to which respondents felt the following in the previous week: (a) calm and peaceful, (b) optimistic about their future, (c) satisfied with their life, (d) happy, (e) anxious (reversed), and (f) depressed (reversed) (Lyubomirsky & Heidi, 1999; Huppert, 2009; Kahneman & Krueger, 2006; Allcott et al., 2020). We also asked respondents about how many days in the previous week they engaged in several unhealthy actions (i.e. order fast food, drink more than one alcoholic beverage a day, getting into an argument) and some healthy ones (i.e. exercise; reverse coded). These two batteries help us analyze the treatment’s effects on various indicators of well-being ($\text {H}_{8a}$ and $\text {H}_{8b}$). These two batteries were also only asked in the post survey.

### Moderators

We use three validated moderators (Huddy et al., 2020) measuring the strength of people’s political attachments. *Party strength:* we fold a 7-point party ID question to create a 4-point party strength measure from Independent to Strong Democrat/Republican. *Ideology strength:* we fold a 11-point ideology scale to create a 6-point ideology strength scale from moderate to extreme liberal/conservative. *Party identity strength:* we average the responses to four questions asking about about how much respondents identify with their political party.

## Limitation

Before presenting the results, we acknowledge that—in maximizing ecological validity by embedding treatments in a larger project and testing boomerang effects in naturalistic settings—we lose some control over treatment. People could choose which article to read, and so different participants may have been experiencing slightly different treatments. We note that our compliance measure includes exposure to articles on *political topics only*, and exploratory analysis of the article topics suggests that the solid majority of the articles read were about contentious and salient issues. Even though some of these issues may not have been those studied in the pre- and post survey, and some participants may have been selecting articles on non-personally involving topics, source cues alone and exposure to articles on the homepages and other headlines may also produce effects (Nicholson, 2011). While this limitation should be kept in mind, we believe the advantages provided by being able to test all the distinct outcomes in an over time experiment with realistic doses of exposure justify the effort.

## Results

### Attitude Polarization

First, we analyze the effects of extremely counter-attitudinal exposure on attitude and affective polarization, and then explore the other effects. We conclude by examining how respondents valued the news outlets to which they were exposed and by discussing the implications of our findings.

**Fig. 3:**
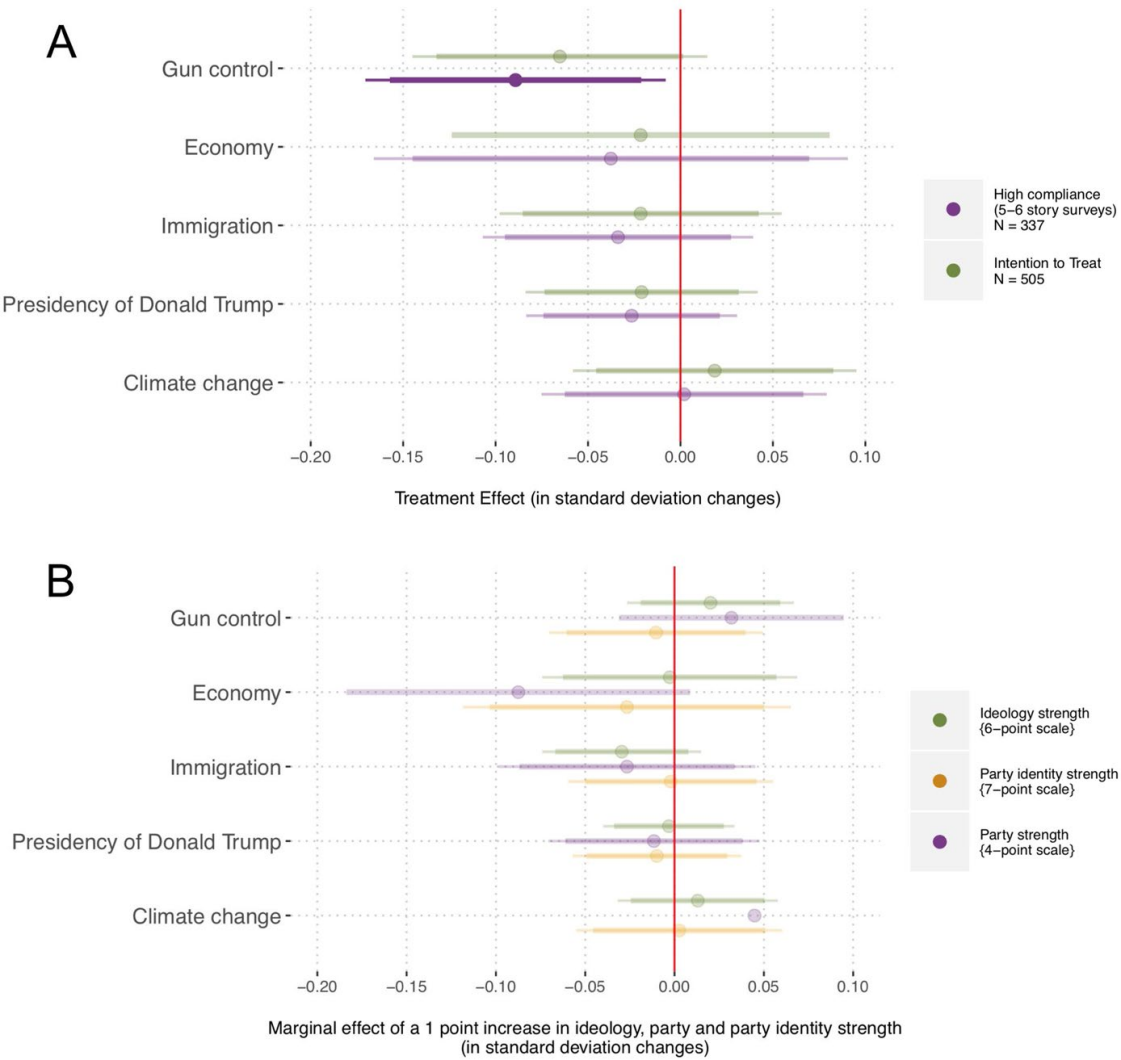
**A** Linear models predicting pre- to post-test changes in issue positions as a function of assignment to treatment. **B** Linear models predicting these changes, as a function of an interaction between assignment to treatment and each moderator. The bars indicate 95 and 90% confidence intervals

In Fig. 3A we report post-treatment differences in attitude polarization between the treated respondents versus control for the five issues, controlling for pre-treatment values. The responses to the policy items were recoded so that higher values indicated more extreme positions for both the liberal and the conservative participants (recall that we did not hypothesize any heterogeneous effects based on partisanship).^11^ We then calculated individual-level post treatment differences and estimated treatment effects by fitting a linear model with assignment to treatment as a single covariate.^12^ In Fig. 3 we report whether those exposed to extreme sites of the opposing ideology radicalized (higher values) or moderated (lower values) their issue positions, compared to the control. Across the models, following the strategy used in similar publications (Bail et al., 2018; Levy, 2021), we report Intention To Treat (ITT) estimates (*Assigned to Treatment* panel) as well as Casual Average Compliance Effects (CACE) for the respondents who more clearly complied with the treatment (completed at least 5 of the 6 story surveys).^13^

The ITT estimates in Fig. 3A indicate that those *Assigned to Treatment* did not polarize their attitudes on any of the policies (economy, climate change, immigration, gun policy, and Presidency of Donald Trump). Looking at those who complied with the treatment (*High Compliance* estimates), we also observe no evidence of attitude polarization. In fact, those who most often visited the extreme sites of the opposing ideology (high compliers) moderated their views on gun control (see also Levy (2021)).^14^ Overall, the ITT estimates and the CACE for high compliers corroborate ${H}_{1a}$: participants’ issue attitudes did not become more extreme.

Although we did not expect people’s attitudes to polarize in the aggregate, we did hypothesize boomerang effects among those with stronger political predispositions (${H}_{1b}$), namely *Ideology Strength*, *Party Strength*, and *Party Identity Strength*. Figure 3B shows the results of three linear models predicting attitude polarization as a function of an interaction between assignment to treatment and each moderator. We fit the model with data from all respondents assigned to treatment. We do not find any support for our hypothesis ${H}_{1b}$ in any of the three moderator models. In sum, the results in Fig. 3 strongly align with the argument that boomerang attitude-extremity effects are the exception rather than the norm (Guess & Coppock, 2018), even in situations when those with strong priors see content from extreme news sites of the opposing ideology.

### Affective Polarization

**Fig. 4:**
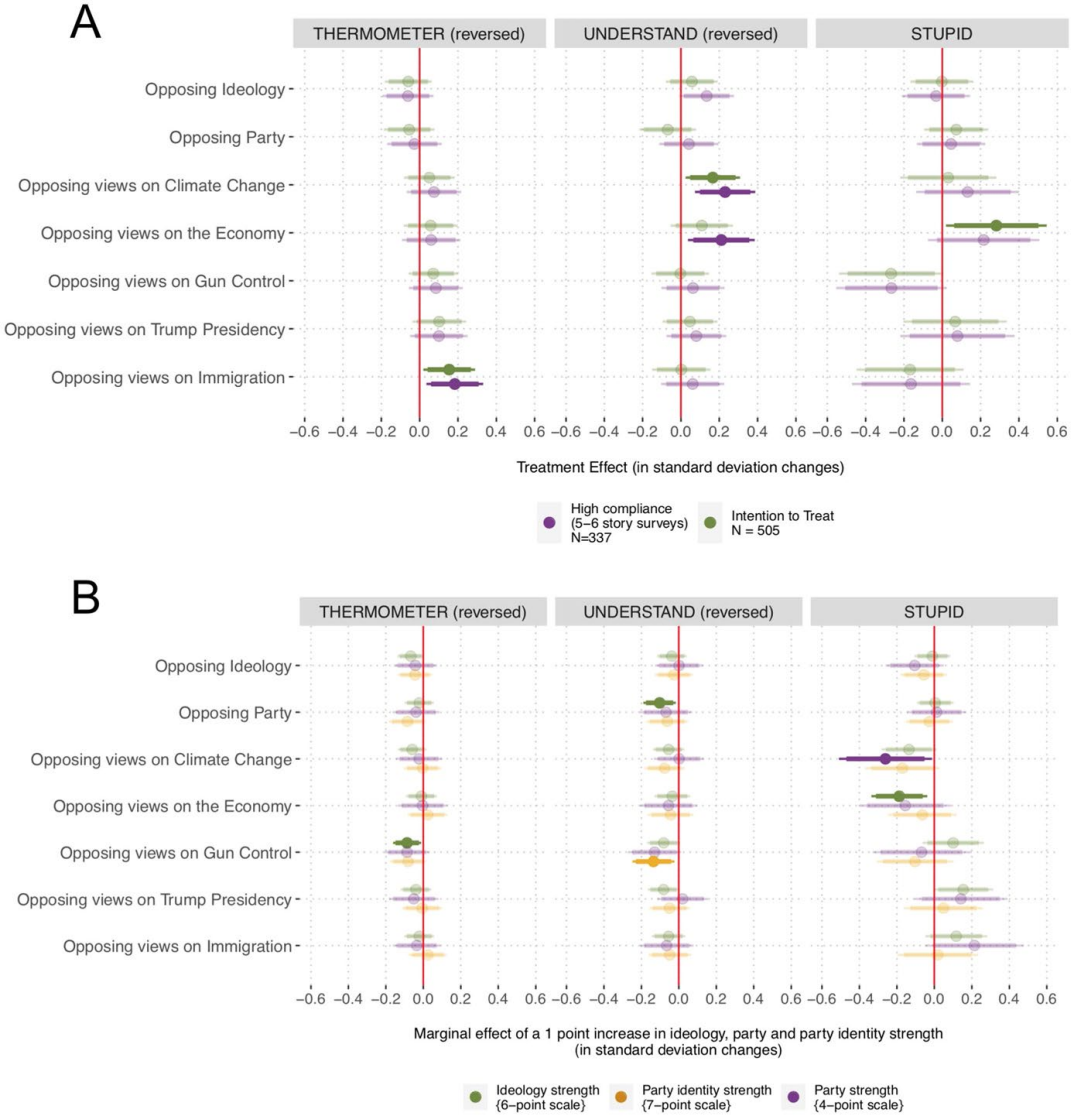
**A** Linear models predicting pre- to post-test changes in affective polarization as a function of assignment to treatment. **B** Linear models predicting these changes as a function of an interaction between assignment to treatment and each of the moderators. The bars indicate 95 and 90% confidence intervals

In Fig. 4 we use the same approach to explore the effects on affective polarization as a function of the treatment. We evaluate changes in feeling thermometers (left panel), understanding (middle panel), and trait ratings (right panel). The *feeling thermometer* and *understand* measures are reversed so that higher values indicate greater affective polarization. To offer comprehensive evidence, we assess affective polarization toward those of the opposing ideology, opposing party, and those who hold different views on the five issues.

Figure 4 clearly shows that, independently of which indicator we examine, people did *not* become more negative towards members of the opposing ideology nor those of the opposing party (no significant positive difference in the first two rows of any of the three panels). However, the treated participants became more negative toward those who opposed their views on several of the issues studied. For example, according to the ITT estimates and the CACEs for high compliers, compared to the control, the treated partisans felt colder towards those with different views on immigration, understood less those with opposing opinions on climate change and the economy, and were more likely to believe that those with opposing views on the economy were stupid. We note, however, that these effects are rather small (i.e., 0.1 and 0.3 standard deviation changes) and also likely due to chance (i.e., they disappear when we adjust for multiple comparisons using the False Discovery Rate (FDR) method (Benjamini & Hochberg, 1995). Overall, we find no support for ${H}_{2a}$.

In Fig. 4B we find no support for the expectation that consuming extreme sites of the opposing ideology would make participants with stronger political identities especially likely to affectively polarize (${H}_{2b}$). None of the coefficients are positive or statistically significant. In fact, contrary to the hypothesis, for some of the indicators, those with stronger identities became *less* affectively polarized. Again, however, this effect disappears when accounting for multiple comparison.

### Further Effects

**Fig. 5:**
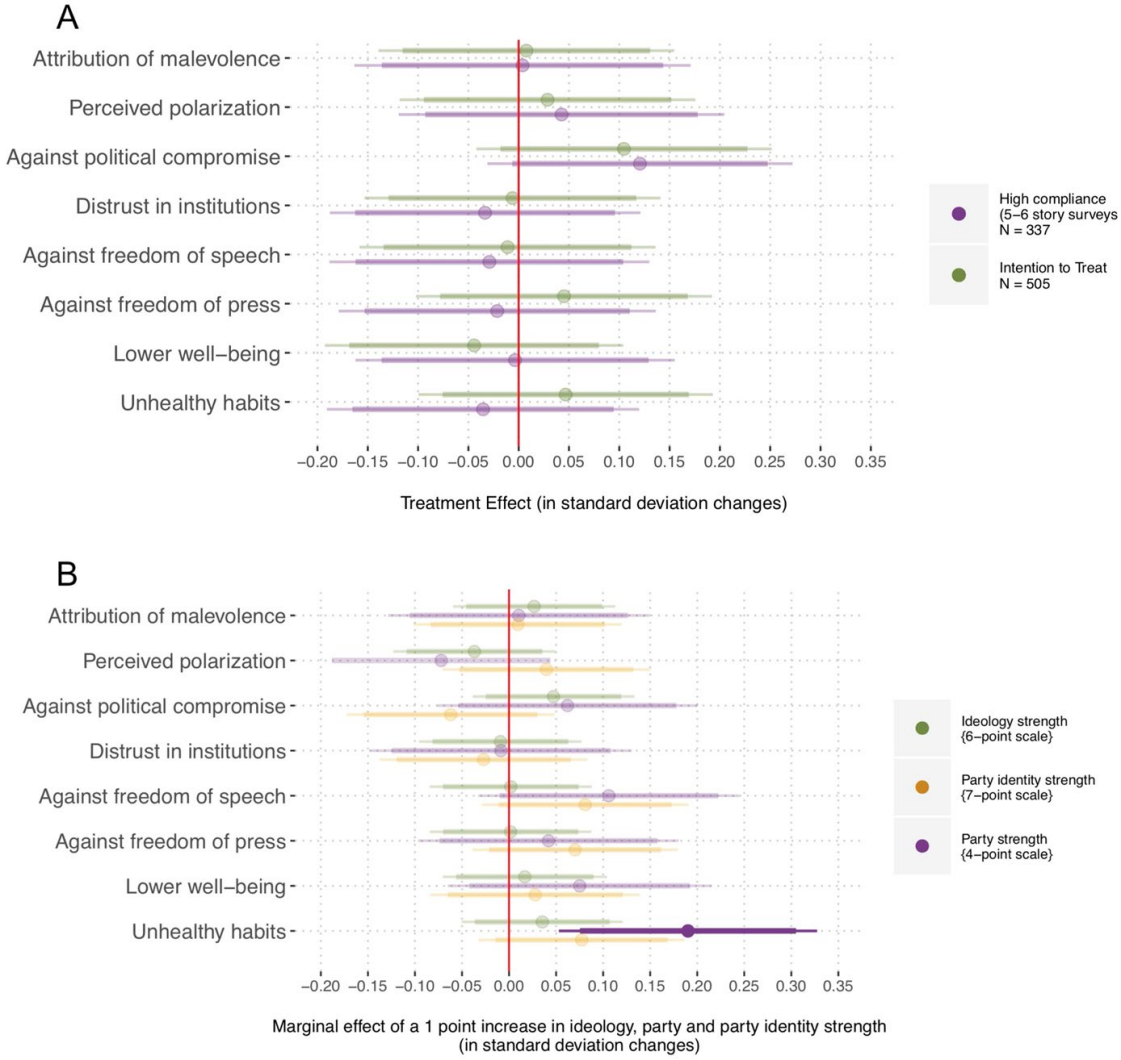
**A** Linear models estimating the effect of exposure to extreme news sites of the opposing ideology on other outcomes. **B** Linear models predicting the same outcomes, as a function of an interaction of assignment to treatment and each moderator. The bars indicate 95 and 90% confidence intervals

In Fig. 5A we report treatment effects for the remaining outcomes of interest. The first three rows show outcomes related to polarization: whether people (a) think out-partisans want to harm the country (*Attribution of malevolance*), (b) perceive the political climate as polarized (*Perceived polarization*), and (c) oppose politicians crossing the aisle and reaching compromises (*Against* *political compromise*). Then, we also report differences on how much participants trust key institutions, whether they are against freedom of speech and freedom of press, and whether they reported declining well-being and undertaking unhealthy activities at a higher rate. For the first four items, we estimate differences between treatment and control while controlling for pre-test values, and estimate differences between treatment and control for the last four items.

Overall, we do not see in Fig. 5.A that exposure to extreme news sites of the opposing ideology had any meaningful effect on any of these outcomes, showing no support for our hypotheses (${H}_{3a, 4a, 5a, 6a, 7a, 8a}$), and extending past evidence on the rarity of boomerang effects; a null finding that holds when looking at those assigned to the treatment and those who complied.

Figure 5B explores whether this null effect holds some heterogeneity. We do not observe stronger effects for those with higher ideology, party, and party identity strength. Although self-identified strong partisans were more likely undertake unhealthy activities (e.g., drink more or order fast food), this finding does not hold when accounting for multiple comparisons. In sum, the evidence does not support our hypotheses ${H}_{3b, 4b, 5b, 6b, 7b}$ and provides very mild support for ${H}_{8b}$.

### Outlet Evaluation

**Fig. 6:**
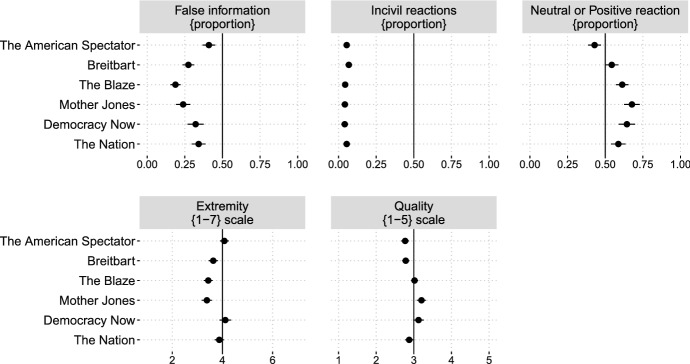
Opinions about and reactions to the extreme news sites to which respondents were exposed

As a final exploratory assessment of the effects, Fig. 6 reports people’s reactions to the outlets and the articles which people read. For one, respondents indicated whether they perceived the outlets to have extreme issue positions and whether they thought the articles they read were of good quality. The two bottom panels in Fig. 6 show that people saw the views of the outlets as rather moderate (*Extremity* panel: an average score between 3 and 4 in a 7-point scale) and of medium quality. Although respondents were able to self-select the politically-relevant articles to read once in the site (as discussed in the Limitations section), we believe that this indicates that despite being exposed to some of the most extreme outlets on the other side, respondents still valued what they saw and mostly did not have negative reactions. Second, the open-ended reactions to each article read were coded as positive, neutral, negative, or mixed, and also as using uncivil language or not.^15^ Shedding important light on the tested effects, people did *not* have an overwhelmingly negative impression of these out-group outlets, as the boomerang effects hypothesis would argue, and as we initially expected. The indicators in the top three panels are proportions. For all six outlets, fewer than 50% of the respondents thought the articles they read provided false or made up information (only between 20–25% for *Breitbart*, *The Blaze*, and *Democracy Now*). Fewer than 10% used uncivil language when writing a reaction to the article they read, e.g., “...I felt irritated that crap like this is even given a platform and audience.” In all cases except *The American Spectator*, over 50% of the respondents wrote a neutral or positive reaction, such as (from a Democrat):“It was a good exercise in open-mindedness for me. I chose it [the article] because I like and respect Ben Stein and was genuinely curious about his topic. It was moving as he recounted the heroes of WWII and then explained about China’s advances/Trump’s policies. While he may be ascribing better intelligence and motives to Trump, I still took away a new grain of respect for Trump, just in case Stein’s understanding is correct.”,^16^ or (from a Republican) “Even as a Republican, I agree with the tax credit for housing when it costs more than 30% of a family’s income.”^17^

## Discussion

In the US, political understanding is needed more than ever. To achieve this elusive goal, scholars and practitioners encourage exposure to dissimilar political views, with the hope that encountering views that challenge one’s beliefs will minimize extremity and interparty hostility. Although some scholars caution against this approach, suggesting that cross-cutting exposure can increase polarization, the evidence of such boomerang effects is mixed and quite limited in scope.

We set out to contribute solving this debate with an innovative experimental design combining incentivized over time exposure to extreme news domains from across the aisle (Breitbart, The American Spectator, and The Blaze for liberals; and Mother Jones, Democracy Now, and The Nation for conservatives), pre-, post-, and intermediate surveys, trace data on actual online exposure, and participants’s open-ended reactions to the outlets tested. Although this design is counterfactual (after all, most liberals are unlikely to regularly visit Breitbart), it was well suited to detecting boomerang effects if these are in fact a likely outcome of cross-cutting exposure. The design also allowed us to test whether the studied exposure impacts broader societal outcomes and individual well-being, and also for whom these effects emerge.

In short, despite the over-time nature of the treatment (i.e., 12 days), accounting for intended treatment effects as well as the levels of compliance, and testing attitude polarization on a range of salient issues and affective polarization with several indicators and toward various out-groups, we show that cross-cutting exposure is unlikely to intensify political conflict or have any substantive effects on the societal and individual outcomes tested. People did *not* radicalize their issue attitudes nor their feelings towards the out-party and the supporters of the opposing ideology. Although we did find that people became slightly more negative toward those holding opposing views on a few policies (e.g., climate change and immigration), these effects were minor (< 0.2 standard deviation changes) and did not hold when accounting for multiple comparisons and false discovery rate. Furthermore, although observers fear that strong partisans are most likely to radicalize and drive political conflict, we do not find pronounced heterogeneous effects.

Similarly, our treatment did little to influence participants’ perceptions of the political system, in terms of their support for compromise, attributing malevolent intentions to the outparty, or seeing the polity as polarized. It also did not shift their support for key democratic principles, such as freedom of speech or freedom of the press. Relatedly, extreme cross-cutting exposure did not worsen participants’ well-being.

The findings are a great contribution to the existing literature on the potential negative effects of exposure to counter-attitudinal information. Contrary to some evidence, which finds exposure to opposing views to exacerbate polarization (Levendusky, 2013; Bail et al., 2018; Garrett et al., 2014), and in line with other recent work (Guess & Coppock, 2018; Guess et al., 2021; Levy, 2021), we conclude that boomerang effect are the exception rather than the norm. Extending past work by incorporating people’s evaluations of the outlets and articles (based on short surveys and also open-ended thoughts and emotions), this consistent lack of boomerang effects may be due to people’s largely neutral or even positive reactions to the outlets and their content. We wanted to test the effect of a counterfactual and selected these 6 sites because they are considered far left and far right in most classifications of news media ideology (Robertson et al., 2018; Eady et al., 2019). Nevertheless, despite representing the extreme of each ideological side,^18^ and despite being vilified by one’s partisan group, our participants sometimes *valued* the information they consumed therein. In addition, this study also makes a relevant contribution to the growing body of work that uses trace data to study people’s attitudes and behavior (Stier et al., 2020; Guess, 2021; Guess et al., 2021; Wojcieszak et al., 2021b). Rather than relying on a forced exposure experiment that shows people mock sites with counter-attitudinal articles, we incentivized exposure, accounted for compliance, and exposed them to real articles that actually appeared in news outlets of the opposing ideology. At a time where key stakeholders such as social media companies (Farr and dorsey, 2018; Wood & Ethan, 2020), news organizations (Goodman & Chen, 2010), and governments (Rendall, 2015; Commission, 2013) are designing policies to reduce polarization, we believe that the findings reported here can help inform the decision-making process moving forward.

## Figures and Tables

**Fig. 7:**
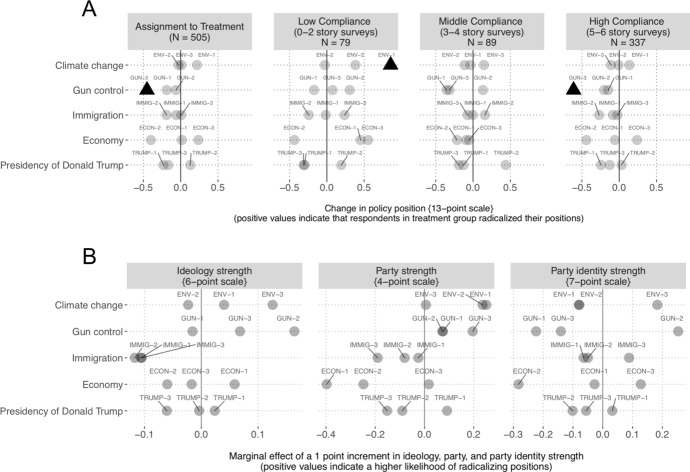
**A** Linear models predicting changes in issue positions between pre and post test, as a function of assignment to treatment. **B** Linear models predicting the same issue position changes, as a function of an interaction between assignment to treatment and each theorized moderator. *Note:* $\bigtriangleup s$ *indicate p-value* $< 0.05$; $\bigcirc$ *otherwise. The three statistically significant results in Panel A are* ***not*** *significant when we account for multiple comparisons* (Benjamini & Hochberg, 1995)

**Fig. 8:**
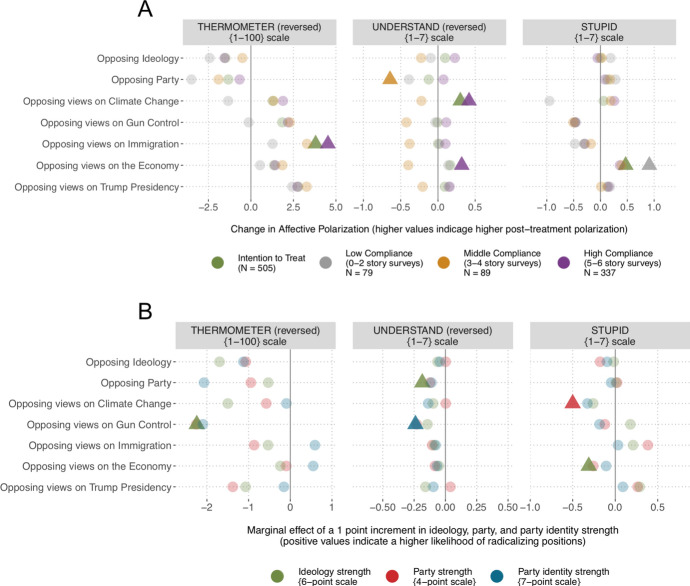
**A** Linear models predicting changes in affective polarization between pre and post test, as a function of assignment to treatment.**B** Linear models predicting the same changes in affective polarization, as a function of an interaction between assignment to treatment and each of the theorized moderators. *Note:* $\bigtriangleup s$ *indicate p-value* $< 0.05$; $\bigcirc$ *otherwise.* ***None*** *of the statistically significant results in Panel A and B remain significant after accounting for multiple comparisons* (Benjamini & Hochberg, 1995)

**Fig. 9:**
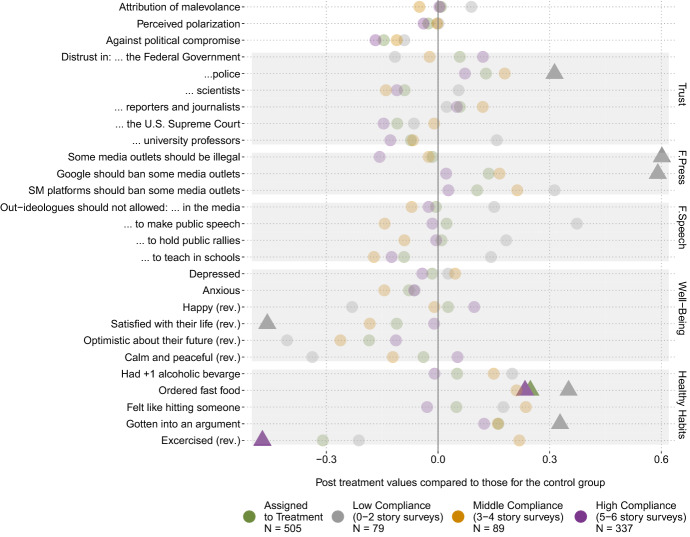
Linear models estimating the effect of exposure to extreme news sites of the opposing ideology on a variety of outcomes relevant for the understanding of political polarization. **B** Linear models predicting the same outcomes, as a function of an interaction of assignment to treatment and each theorized moderator. *Note:* $\bigtriangleup s$ *indicate p-value* $< 0.05$; $\bigcirc$ *otherwise.* ***None*** *of the statistically significant results remain significant after accounting for multiple comparisons* (Benjamini & Hochberg, 1995)

**Fig. 10:**
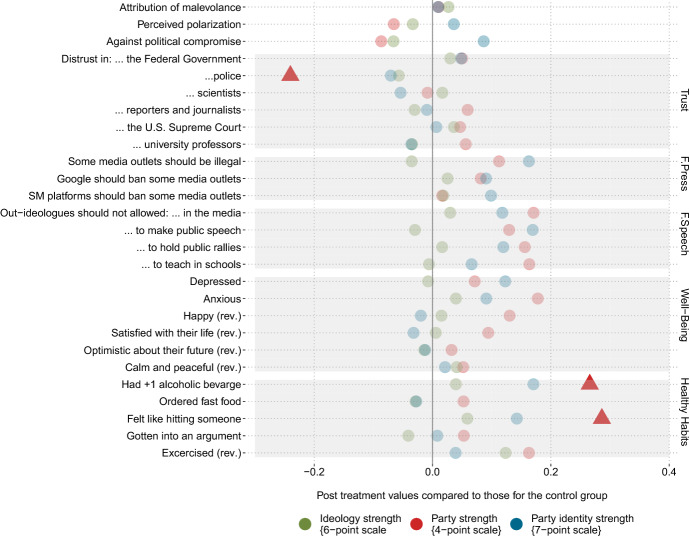
Linear models predicting the same outcomes in Figure 9, as a function of an interaction of assignment to treatment and each theorized moderator. *Note:* $\bigtriangleup s$ *indicate p-value* $< 0.05$; $\bigcirc$ *otherwise*. ***None*** *of the statistically significant results remain significant after accounting for multiple comparisons* (Benjamini & Hochberg, 1995)

**Fig. 11:**
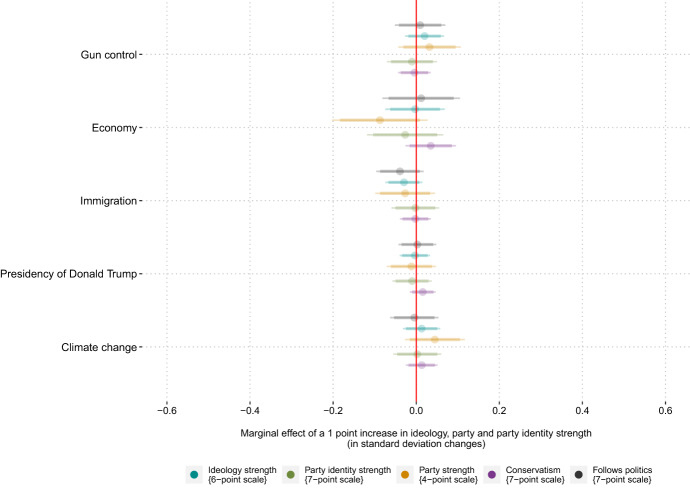
Linear models predicting changes in issue positions, as a function of an interaction between assignment to treatment and each moderator. The bars indicate 95 and 90% confidence intervals

**Fig. 12:**
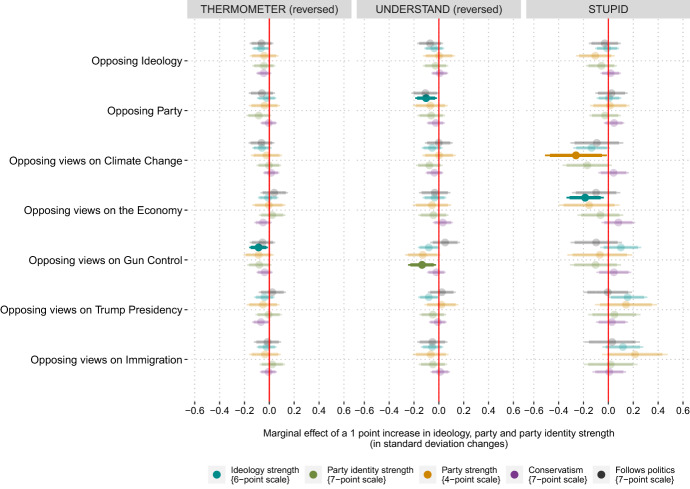
Linear models predicting the changes in affective polarization, as a function of an interaction between assignment to treatment and each of the moderators. The bars indicate 95 and 90% confidence intervals. *Note* ***None*** *of the statistically significant results remain significant after accounting for multiple comparisons* (Benjamini & Hochberg, 1995)

## Appendix 1: Policy Attitudes Questionnaire

On the scales below, please indicate whether your opinion is closer to the sentence on the left or the sentence on the right. If you are in the middle, don’t know or are undecided, please chose the middle option. There are no wrong or right answers, we want to know what you think.

(13-point scales)

First, we have questions about the *economy*.

**Table:**

Economy 1	Government regulation of business is necessary to protect the public interest	Government regulation of business usually does more harm than good
Economy 2	Government should raise taxes to increase public services	Government should cut public services to cut taxes
Economy 3	Free trade has harmed the U.S. economy	Free trade has helped the U.S. economy

Now we have questions about *climate change and the environment*:

**Table:**

Climate 1	Stricter environmental laws and regulations are worth the cost	Stricter environmental laws and regulations cost too many jobs and hurt the economy
Climate 2	There is solid evidence of the global warming caused by human activity	There is no solid evidence of global warming caused by human activity
Climate 3	To solve the nation’s energy problems, the US should emphasize the development of alternative energy, such as wind and solar power	To solve the nation’s energy problems, the US should emphasize the production of oil, gas and coal supplies

Now we have questions about *immigration*:

**Table:**

Immigration 1	Immigrants today strengthen our country because of their hard work and talents	Immigrants today are a burden on our country because they take our jobs, housing, and healthcare
Immigration 2	Government should allow unauthorized immigrants to remain in the United States and eventually qualify for U.S. citizenship, without penalties	Government should make all unauthorized immigrants felons and send them back to their home country
Immigration 3	American identity, norms and values have been enriched thanks to the presence of immigrants.	American identity, norms and values are being threatened because there are too many immigrants in the US.

Now we have questions about *gun control*:

**Table:**

Gun Control 1	The federal government should make it more difficult to buy a gun than it is now	The federal government should make it easier to buy a gun than it is now
Gun Control 2	Banning the sale of semi-automatic weapons will prevent mass shootings	Banning the sale of semi-automatic weapons will do nothing to prevent mass shootings
Gun Control 3	Carrying a concealed gun should not be allowed anywhere	Carrying a concealed gun should be allowed everywhere

Now we have questions about *the presidency of Donald Trump*:

**Table:**

Trump 1	Generally, I trust what Donald Trump says LESS than I trusted what previous presidents said while in office	Generally, I trust what Donald Trump says LESS than I trusted what previous presidents said while in office
Trump 2	Trump respects white people and men much more than he respects women and minorities	Trump respects all social groups equally, cares for people like me
Trump 3	Trump’s presidency has been bad for the economy	Trump’s presidency has been good for economy

## Appendix 2: Measures: Additional Information

### Question Wording

#### Attribution of Malevolance

**Table:**

Rows	Columns
1. I worry that [Opposite Party] are deliberately trying to hurt America	1. Strongly disagree
2. [Opposite Party] are knowingly sabotaging the country	2.
3. [Opposite Party] don’t care about America	3.
4. I believe [Opposite Party] genuinely wants what is best for America*	4. Neither
5. I trust [Opposite Party] to do what they think is best for America*	5.
	6.
	7. Strongly agree

Note: **reversed items*

#### Support for Compromise

**Table:**

Left statement	Right Statment
1. Politicians need to hold to their principles no matter what	1. Politicians need to work together to get things done
2. Politicians should never compromise their values	2. Sometimes compromise is necessary when addressing major problems
3. I want politicians who hold their ground	3. I want politicians who work together
4. Principles should never be compromised	4. Principles should never block progress

#### Perceived Polarization

**Table:**

Rows	Columns
1. Democrats and Republicans hate each other	1. Strongly disagree
2. The differences between Democrats and Republicans are too great to be reconciled	2.
3. Americans are greatly divided when it comes to the most important values	3.
4. Polarization in America is greater than ever before	4. Neither
	5.
	6.
	7. Strongly agree

#### Trust

**Table:**

Rows	Columns
1. The federal government in Washington	1. Not trust at all
2. Police	2.
3. Scientists	3.
4. Reporter and journalists	4. Moderately
5. The U.S. Supreme Court	5.
6. University professors	6.
	7. Completely trust

#### Political Identity Strength

**Table:**

Rows	Columns
1. I often think of myself as a [Selected Party]	1. Strongly disagree
2. I consider myself a typical [Selected Party]	2. Disagree
3. I’m proud that I’m a [Selected Party]	3. Somewhat disagree
4. If someone said something bad about [Selected Party], I feel as if they said something bad about me	4. Neither agree nor disagree
	5. Somewhat agree
	6. Agree
	7. Strongly agree

### Cronbach’s Alpha for all Constructed Indeces

See Table 2.

**Table 2:** Cronbach alphas assessing the reliability of the indices created for this study, as well as average values and standard deviations

Index	Average	Standard	Cronbach’s
		Deviation	Alpha
Attitude: Gun control	**5.62**	**3.58**	**0.87**
Attitude: Economy	**7.20**	**2.40**	**0.48**
Attitude: Immigration	**5.74**	**3.69**	**0.92**
Attitude: Climate change	**5.74**	**3.69**	**0.92**
Attitude: Trump’s presidency	**5.42**	**4.35**	**0.95**
Political identity strength	**4.80**	**1.34**	**0.84**
Attribution of malevolance	**4.66**	**1.51**	**0.87**
Perceived polarization	**4.72**	**1.11**	**0.70**
Against political compromise	**4.99**	**1.53**	**0.89**
Trust in institutions	**4.28**	**1.05**	**0.76**
Freedom of speech	5.35	1.46	0.94
Freedom of the press	3.63	1.67	0.82
Well-being	2.48	1.32	0.87
Unhealthy habits	-0.01	1.01	0.54

Bold rows provide numbers based on data from the pre-survey, while the remainin rows provide data based on the post-survey (these questions were not asked in the pre-survey)

## Appendix 3: Finer-Grained Results

For the sake of simplicity, in the main analysis we aggregated several results. For example, when exploring changes in attitudes towards our five issues of interest (gun control, economy, immigration, presidency of Donald Trump, and climate change), we aggregated the respondents’ policy positions on three sub-issue dimensions for each of the issues. Moreover, in the main analysis we did not report results for those who barely/moderately complied with the treatment. In this Appendix we include a more desegregated and complete version of Figs. 3, 4, and 5. Compared to the figures in the main text, where the results are expressed in standard deviation changes, note that in here we report changes in the scale of the outcome variable (Figs. 7, 8, 9, 10).

## Appendix 4: Moderator Analysis Including Party ID and Interest-in/Following politics

Some research finds boomerang effects among Republicans/conservatives but not among Democrats/liberals (Bail et al., 2018). We did not hypothesize heterogeneous effects based on partisanship/ideology and so we did not include it as key moderator in the main analysis. However, in this Appendix we include a 7-point self-reported variable measuring partisanship (*Conservatism*, ranging from *Strong Democrat* to *Strong Republican*) to our moderator analysis for attitude and affective polarization. In addition, previous analysis indicate that most people do not consume news (Arceneaux & Johnson, 2013) yet a small group of the population are strong news consumers and follow politics closely (Castro et al., 2021). In this Appendix we also assess whether boomerang effects emerge among those who follow politics more intensively, by including as moderator a 7-point self-reported variable measuring how closely people follow news (measured in Wave 1 of the 3-wave panel study). We do not observe these two additional moderators to have any effect (Figs. 11, 12).

## Appendix 5: Sample Descriptives

See Table 3.

**Table 3:** Pre-test values for all respondents who completed the pre-test, for those who accepted to participate in the experiment, for those who completed each story survey, and for those who completed the post test

	Compl.	Accepted	Story	Story	Story	Story	Story	Story	Post	Treat.	High	Cntrl
	Wave 2	Particip.	Survey 1	Survey 2	Survey 3	Survey 4	Survey 5	Survey 6	Survey	Group	Compl.	Group
Age (avg.)	44.4 (14.6)	44.5 (14.6)	43.3 (14.6)	43.7 (14.5)	43.4 (14.4)	43.2 (14.3)	44 (14.5)	43.9 (14.6)	44.8 (14.4)	45 (14.8)	43.5 (14.6)	44.3 (13.8)
Female (%)	56.7	56.9	54.8	55.4	54	53.6	54.2	55.2	57	55.5	50.3	59.9
Education: no high school (%)	0.1	0.1	0	0	0	0	0	0	0.1	0.2	0	0
Education: high school (%)	12.9	12.7	11.9	12	12.5	12.2	11.9	11.6	13	12.4	11.7	14.1
Education: college (%)	65.4	65.9	65.7	65.1	64.3	64.1	64.7	65.8	64.9	63.9	64.7	66.8
Education: Graduate studies (%)	20.7	20.3	21.7	21.9	22.2	22.7	22.6	21.5	20.9	22.3	23	18.3
Ethnicity: White (%)	80.1	80.2	78.8	80	79	79.3	79.9	78.7	80.3	79.2	78.2	82.4
Ethnicity: Black (%)	10	9.9	9.6	9.2	9.3	9.5	9	9.7	10	10.5	9.5	9.2
Ethnicity: Asian (%)	5.5	5.4	6.8	6.2	7.3	6.7	6.7	6.7	5	5.8	7.1	3.4
Ethnicity: Native American (%)	1.2	1.3	1.2	1.2	0.7	0.7	1.2	1	1.3	1	0.9	1.9
Ethnicity: Hispanic (%)	11.5	11.7	11.9	12.8	11.8	12	11.5	11.9	12.1	11	12.9	14.2
Party ID: Strong Democrat (%)	25	24.5	23.5	25.1	25.4	26	26.2	24.7	24.3	24.2	25.8	24.5
Party ID: weak Democrat (%)	14.3	14	14.3	14.6	14	14.7	13.9	15	13.9	14.1	15.1	13.7
Party ID: independent leaning Democrat (%)	14.8	15	17	16.5	15.4	14.7	16.3	15.7	15.2	15.8	16	14
Party ID: independent (%)	12.7	12.9	13.2	13.8	13.3	13.5	13.5	13.3	13.2	13.3	14.5	12.9
Party ID: independent leaning Republican (%)	8.7	8.2	8.7	8.4	8.8	8.2	9.1	9.3	8	7.9	8.6	8.3
Party ID: weak Republican (%)	11	11.4	9.6	9.8	9	10.4	9.4	9.5	11.4	11.1	7.7	11.9
Party ID: strong Republican (%)	13.5	13.9	13.5	11.9	14.2	12.5	11.5	12.6	14	13.7	12.2	14.7
Party Identity Strength {1–7} (avg.)	4.8 (1.3)	4.8 (1.3)	4.7 (1.3)	4.7 (1.3)	4.7 (1.3)	4.7 (1.3)	4.7 (1.3)	4.7 (1.3)	4.8 (1.3)	4.7 (1.3)	4.7 (1.3)	4.9 (1.3)
Conservatism {0–10} (avg.)	4.8 (3.1)	4.8 (3.1)	4.6 (3)	4.5 (3)	4.7 (3.1)	4.5 (3.1)	4.5 (3)	4.6 (3.1)	4.8 (3.1)	4.7 (3.1)	4.5 (3)	5 (3)
Feeling Therm {0–100}: Out-party (avg.)	27.5 (24.8)	27.2 (24.7)	27.5 (24.6)	27.3 (23.7)	26.8 (23.3)	27.3 (23.9)	27.2 (23.9)	27.5 (23.6)	27.4 (24.7)	27.3 (23.9)	27.5 (23.8)	27.4 (26.3)
Feeling Therm {0–100}: Out-ideology (avg.)	31.4 (24.7)	31.2 (24.7)	30.6 (24.6)	30.6 (24.3)	30.3 (23.7)	30.7 (24.4)	30.7 (24.3)	30 (23.6)	31.2 (24.8)	30.6 (24)	30.6 (23.9)	32.2 (26.1)
Issue Position {1–13}: Economy (avg.)	7.2 (2.4)	7.2 (2.4)	7.2 (2.3)	7.1 (2.3)	7.2 (2.4)	7.2 (2.4)	7.1 (2.3)	7.2 (2.3)	7.2 (2.4)	7.2 (2.4)	7.1 (2.3)	7.1 (2.4)
Issue Position {1–13}: Immigration (avg.)	5.8 (3.7)	5.8 (3.7)	5.5 (3.6)	5.3 (3.6)	5.4 (3.6)	5.3 (3.5)	5.2 (3.5)	5.3 (3.5)	5.7 (3.7)	5.6 (3.6)	5.2 (3.5)	5.9 (3.8)
Issue Position {1–13}: Climate Change (avg.)	4.7 (3.6)	4.7 (3.6)	4.6 (3.6)	4.4 (3.5)	4.6 (3.6)	4.5 (3.6)	4.4 (3.5)	4.5 (3.6)	4.8 (3.7)	4.8 (3.6)	4.4 (3.6)	4.9 (3.7)
Issue Position {1–13}: Gun Control (avg.)	5.6 (3.6)	5.6 (3.6)	5.6 (3.6)	5.2 (3.5)	5.5 (3.6)	5.3 (3.5)	5.3 (3.5)	5.5 (3.6)	5.6 (3.6)	5.6 (3.6)	5.3 (3.5)	5.6 (3.6)
Issue Position {1–13}: Trump’s presidency (avg.)	5.4 (4.3)	5.4 (4.3)	5.2 (4.2)	4.9 (4.1)	5.2 (4.2)	5 (4.2)	4.9 (4.1)	5.1 (4.2)	5.4 (4.4)	5.3 (4.3)	4.9 (4.1)	5.6 (4.5)
Feeling Therm {0–100}: Economy (avg.)	38.6 (23.6)	38.3 (23.7)	38.3 (23)	37.3 (22.1)	37.4 (22.9)	37.4 (22.9)	37.7 (22.9)	37.8 (22.5)	38 (23.7)	37.8 (23.1)	37.3 (23.3)	38.3 (24.7)
Feeling Therm {0–100}: Immigration (avg.)	31.2 (24.8)	31.2 (24.8)	31.3 (25.2)	30.8 (24.5)	30.9 (24.3)	31.4 (24.6)	30.6 (24.8)	30.6 (24.4)	30.8 (24.6)	30.9 (24.4)	30.7 (24.6)	30.5 (25)
Feeling Therm {0–100}: Environment (avg.)	29.6 (25.3)	29.7 (25.3)	29.5 (25.3)	28.1 (24.8)	28.4 (24.9)	28.4 (25)	27.8 (24.7)	27.6 (24.6)	29.6 (25.2)	29.1 (25)	27.5 (24.9)	30.7 (25.6)
Feeling Therm {0–100}: Gun Control (avg.)	29.2 (26)	29.3 (26.1)	29.6 (25.7)	29.2 (25.4)	29.4 (25.6)	29.5 (25.7)	28.4 (25.2)	28.9 (25.1)	29.2 (25.8)	30 (25.5)	28.5 (25.4)	28 (26.5)
Feeling Therm {0–100}: Trump’s presidency (avg.)	26.8 (27.4)	26.6 (27.2)	26.9 (27.1)	26.8 (27.4)	26.6 (26.6)	26.2 (26.6)	26 (26.8)	25.5 (25.7)	26.3 (27.2)	27 (26.9)	25.9 (26.6)	25 (27.7)
N	1029	958	446	419	422	415	416	421	783	505	337	278

For binary/categorical variables, we indicate the percentage of respondents belonging to each group. For continuous variables, we provide average values as well as standard deviations in parenthesis. All numbers in the Table have been rounded to 1 decimal number

## Appendix 6: Random Sample of Reactions to Treatment Articles

In this Appendix we illustrate more clearly the kinds of news articles to which respondents were experimentally exposed, as well as the reactions they wrote after reading them. We randomly selected them by setting the random seed only once (value: 123, we did not try any additional seed value) and pulling 20 responses from the whole sample.

**Table:**

1. Response from a Liberal to this *article* from Breitbart:	
While I felt that the article did contain certain facts, I also thought that there were quite a few things that were completely open to interpretation, and I think that there were too many items that were two or three-word segments that were cut from longer sentences that the writer had placed in to fill their own representation of events	
2. Response from a Liberal to this *article* from Breitbart:	
Democrats Can’t Deport 11 Million of Illegals, but Will Confiscate 16 Million AR-15s...bottom line is the immigrants is not what is a huge problem in this country, its the guns that are killing our children. White man/young men with ar 15’s are what we need to worry about	
3. Response from a Conservative to this *article* from The Nation:	
I found the article to be a waste of time with it only giving vague details about climate change politics. Not once does the article go into detail on its points and relied heavily on insulting other people without even giving any reason why these people don’t believe in climate change. I for one believe climate change exists, but that means nothing if I can’t back it up with facts. All in all, I felt the article was solely made to make money on advertisements	
4. Response from a Conservative to this *article* from Mother Jones:	
Democrats are investigating Vice President Pence’s visit to Ireland. Since he visited Trump’s property while in Ireland. Dems are afraid that too much money was spent on this visit	
5. Response from a Conservative to this *article* from Democracy Now:	
I think it’s a good article because it have a lot of useful information.	
6. Response from a Liberal to this *article* from The American Spectator:	
I felt mellow. It made me wonder what can we do early in life to try to combat this problem	
7. Response from a Conservative to this *article* from Mother Jones:	
I do not think that we have a gun problem in the US. I do think that there is a mental illness crisis in the US	
8. Response from a Conservative to this *article* from The Nation:	
Climate change has to happen to save our planet and I think the article shows some good points and solutions	
9. Response from a Conservative to this *article* from The Nation:	
I think one of the most prominent American values is the belief that one can rise to the elite through hard work and commitment. The article I just read shows that this belief is very, very wrong, as the system that supposedly promotes this myth, the meritocracy, is doing the opposite; preventing middle and working-class individuals from rising to the top. Even worse, meritocracy is also negatively impacting elites, as fewer and fewer of them are subjected to intense competition and self-exploitation, something that is being passed onto their children. I agree that the meritocracy system needs a complete overhaul via expanding access to elite labor and education	
10. Response from a Conservative to this *article* from The Nation:	
Palestinians are being denied entry to United States	
11. Response from a Liberal to this *article* from The Balze:	
I felt more knowable	
12. Response from a Liberal to this *article* from Breitbart:	
It was a short article, but it wasn’t very informational	
13. Response from a Liberal to this *article* from Breitbart:	
I suspect Trump is not as positive at helping republicans win, when he gives an endorsement.Comments here re: Elizabeth Warren were obvious personal opinions, not facts	
14. Response from a Liberal to this *article* from The Blaze:	
I think that the article shows that there is growing shift on views of certain types of guns after several mass shooting over ht period of several years. It also shows that there are still people who are hardcore gun advocates that thing the slightest thing is a violation of their rights	
15. Response from a Conservative to this *article* from The Nation:	
Its a mix of good and bad for myself between Trump	
16. Response from a Liberal to this *article* from The American Spectator:	
This article was a love letter to David Koch, the billionaire and money-thrower held in high regard by the Republican Party and outspoken conservatives at large.	
17. Response from a Liberal to this *article* from Breitbart:	
I think Prascale is ridiculous. The family does not stand for strong conservative values and I’m embarrassed that it is an article	
18. Response from a Conservative to this *article* from Democracy Now:	
It’s very important article	
19. Response from a Liberal to this *article* from Breitbart:	
This is a bunch of misrepresentations and a few lies. I expected this from Breitbart, and now it did it. There are a majority of democrats supporting impeachment, and some change their minds not because of "politics," but because their constituents are asking for it and supporting it	
20. Response from a Liberal to this *article* from Breitbart:	
That donald trump jr is an idiot	

## Footnotes

1 Numerous studies examine ’backfire effects’ in the context of correcting misinformation, a focus that is distinct from ours, and so we do not engage with this literature; for reviews see Wood and Porter (2019) and Wood and Ethan (2020).

2 https://osf.io/vxqzt/?view_only=11e823483f0c4c8ab73f6897baebaa8d.

3 Even though a fraction of the U.S. population are heavy consumers of partisan media or live in media bubbles (Prior, 2013; Eady et al., 2019), this small group encompasses strong and active partisans, who hold substantive influence over the political sphere (Druckman et al., 2018; Barbera et al., 2019).

4 Respondents were not aware of the nature of the study until they were debriefed at the end.

5 Respondents were assigned to the liberal group (N = 393) if they fulfilled one of the following conditions: they were (a) a Democrat in wave 2 (n = 346, 88% of 393), (b) an Independent in wave 2 but had indicated to be a Democrat in wave 1 (n = 14, 4%), (c) an Independent in wave 1 and 2 but a liberal according to an ideology question in wave 2 (N = 10, 3%), and (d) an Independent in wave 1 and 2, and a moderate in wave 2, but who was imputed to be a Democrat according to a highly accurate 2-layer neural net (87% precision and recall) we trained using the answers to a set of policy issues and psychological traits questions from wave 1 (N = 23, 5%). The remaining respondents to be treated were assigned to the conservative group (N = 247): (a) 211 (85% of 247) indicated they were Republican in wave 1, (b) 8 (3%) said Independent in wave 2 but Republican in wave 1, (c) 14 (6%) said Independent in both waves but had a conservative ideology in wave 2, and (d) 14 (6%) who reported to be Independents and have moderate ideology in both waves but we imputed to be Republicans.

6 You can find a (randomly selected) sample of articles and responses in Sect. 14.

7 Out of the 505 respondents who were assigned in the treatment group and who completed the pre as well as the post survey, we obtained browsing data for 353 (70%).

8 Because the experiment was embedded in a larger project, we did not conduct power analyses a priori for this experiment. We acknowledge growing criticisms of post-hoc power analyses (Hoenig & Heisey, 2001), which instead recommend direct comparisons with previous similar studies. We note that our sample size (505 in treatment group, 279 in control group) is similar to the sample size in some of the most recent papers on this topic, which report small to very small treatment effects (i.e. around .10 standard deviation changes): e.g. Bail et al. (2018) (374 treatment, 244 control for Democrats, and 282 treatment, 169 for Republicans—as the authors compared treatment and control within party); Guess et al. (2021) (361 FoxNews treatment group, 360 HuffPost treatment group, 377 control group).

9 These statements were taken or adjusted from questions asked by ANES, PEW, and Gallup.

10 The Cronbach alpha for the Economy is slightly below 0.6. Thus, in Sect. 11 we report results broken down by sub-issue dimension.

11 See Sect. 12 for exploratory analyses. We find null effects, so Democrats and Republicans as well as conservatives and liberals reacted similarly to our treatment.

12 Random assignment was successful: a linear model predicting assignment to treatment as a function of a set of socio-demographic covariates (age, gender, ethnicity, education, as well as a a measure of interest in politics) generated no statistically significant results.

13 11 presents finer-grained results for this and the following sections; as well as results for those who barely/moderately complied with the treatment.

14 When running multiple comparisons, some effects may be statistically significant only by chance. We adjust the significance tests for multiple comparisons using the common FDR (False Discovery Rate) technique (Benjamini & Hochberg, 1995). When doing so, the one minor significant effect depicted in Fig. 3 cease to be statistically significant at the 0.05 level. Given that the pre-registration plan did not specify the adjustment, for transparency we do no alter the figure. Nevertheless, here and throughout, we note that this effect is not robust and likely due to chance, and so we caution against putting any leverage on them.

15 On average, participants wrote 31 words in reaction to this question (median 21 words). Two trained coders categorized the 2844 reactions with 15% overlap (Cohen’s Kappa 0.84 for sentiment coding; 0.94 for civility.

16 Reaction to https://spectator.org/why-i-pray-for-trump-a-true-hero/.

17 Reaction to https://www.motherjones.com/politics/2019/09/vouchers-tax-credits-zoning-can-a-president-do-anything-to-fix-the-housing-crisis/. In Sect. 14 we provide further example reactions, selected at random.

18 They are not as extreme as some niche sites, and although we could have sent people to Stormfront or Antifa, this would have been normatively problematic.
